# Personalizing product sets to individual health priorities increases the healthfulness of hypothetical food choices in US adults

**DOI:** 10.1038/s41598-025-92784-1

**Published:** 2025-03-07

**Authors:** Christopher R. Gustafson, Henriette Gitungwa, Julie B. Boron, Devin J. Rose

**Affiliations:** 1 Department of Agricultural Economics, University of Nebraska-Lincoln, 314A Filley Hall, Lincoln, NE 68583-0922 USA; 2 Department of Gerontology, University of Nebraska-Omaha, Omaha, NE 68182 USA; 3 Department of Food Science and Technology, University of Nebraska-Lincoln, Lincoln, NE 68588 USA; 4 Department of Agronomy and Horticulture, University of Nebraska-Lincoln, Lincoln, NE 68583 USA; 5 Nebraska Food for Health Center, University of Nebraska-Lincoln, Lincoln, NE 68588 USA

**Keywords:** Human behaviour, Nutrition, Preventive medicine

## Abstract

Recently, the potential for dietary personalization based on genetic/phenotypic characteristics to improve health has been studied. While promising, inputs into this biology-focused personalization process are intensive and may not align with an individual’s own health priorities, which drive health behaviors. Here, we examine how personalizing food suggestions based on individuals’ health priorities affects the healthiness of their food choices. We conducted a pre-registered experiment examining hypothetical food choices from three food categories in six conditions: (1) control, (2) health priority prime, (3) healthy product subset, (4) health priority prime + healthy product subset, (5) health priority prime + priority subset, and (6) health priority-based personalized product suggestions. Participants in conditions 2, 4, 5, and 6 first encountered a question asking them to select their top health priority from a list of options. In conditions 5, the subset of healthy items was described as foods beneficial for the selected health priority, while in condition 6, participants immediately saw the set of foods beneficial for the selected health priority, but had the option to see all foods instead. After making food choices, participants completed a survey with questions about the choice process, health priorities, and demographic variables. We used logistic regression to analyze the impact of condition on healthiness of food choices, and ordered logistic regression to examine the impact of condition on satisfaction with choices made. The experiment and survey were completed by 4171 adults (≥ 19 years) in the US, with the sample closely matching US distribution of age, sex, education, and income characteristics. There were no significant differences in the distribution of demographic characteristics among conditions. All intervention conditions significantly increased the likelihood that an individual chose a healthy food. However, interventions that combined priming with healthy subsets were significantly more effective than single interventions. Conditions that connected the healthy subsets to individuals’ health priorities were particularly effective. The adjusted odds ratio (aOR) of selecting a healthy food was 4.77 (95% CI 4.12, 5.52) relative to the control condition when participants could view a subset described as helpful for their health priority. When people immediately viewed the personalized product set, the aOR increased to 11.67 (95% CI 0.1, 13,5). Likewise, analysis of nutrient content from food choices revealed that personalization decreased saturated fat, added sugar, and sodium and increased dietary fiber, potassium, iron, and calcium. However, product choice satisfaction was significantly lower in the personalized product set, which appears to be partially due to a tendency in this condition to forego choosing a product rather than selecting an unhealthy product. Personalization of product options based on individual health priorities should be tested in real-choice environments.

## Introduction

Evidence of the harmful impact of low-quality diets on bodyweight, morbidity, and mortality has increased markedly in recent years^1–4^, spurring efforts to promote healthier food consumption. Overconsumption of unhealthy foods and low intake of healthy foods, including pulses, whole grains, and fruits and vegetables, is related to increased bodyweight^5^, heightened risks of type-2 diabetes, cancer, cognitive decline, and heart disease^6,7^, and lower life expectancy^3,8^.

Multiple forces may constrain healthy food choices. Limited consumer understanding of the health implications of food choices complicates their abilities to make choices that lead to desired outcomes^9–11^. Furthermore, even if consumers understand general impacts of food choices on health outcomes, there may be non-negligible time and search costs to identifying healthy products. In many food categories, the foods containing attributes that help achieve health outcomes may represent a small percentage of available products^12^. For instance, while pulses are quite healthy, pulse-based foods represent a small percentage of products and are difficult for consumers to identify in retail environments^13–15^. Price and taste are important drivers of food choices^16^. Many people cite the cost of healthy foods as a barrier to healthy eating^17–21^, and multiple studies find links between healthy foods and higher prices^22–24^.

The complexity of the retail environment itself may crowd out efforts to make healthy choices. There is evidence that increasing the number of products people must consider causally decreases attention to product attribute information^25^. Consumers frequently consider only a small percentage of products while shopping^26,27^. Further, awareness of policies or interventions to increase healthy food choice may be limited—relatively low percentages of people reported (1) knowledge of taxes in countries with taxes on sugar-sweetened beverages^28^, which may limit their effectiveness^29,30^, (2) awareness of national public health campaigns^31^, (3) noticing calorie information in restaurants^32^, and (4) viewing new nutrition labels^33^, which may partially explain markedly smaller estimated impacts from these policies upon implementation in retail environments than when tested in experiments^34^.

While informational and price-based strategies have had limited impact on the nutritional quality of food choices^35–40^, the rise of online shopping^41,42^ and increasing pervasiveness of smart phones^43^ present opportunities to develop and test new interventions. A systematic review of personalized dietary suggestions, a popular recent strategy to promote healthy choices featuring personalization of food recommendations based on genetic or phenotypic needs, found significant, positive impacts from personalization of dietary recommendations^44^. However, these personalization processes require intensive inputs, such as comprehensive information about diet, genetic, or phenotypic profiles on an individual basis, and may not align with individuals’ perceptions of their own health status, which influences diet and exercise behaviors^45–47^. Other forms of personalization exist that might be less informationally intensive. These include food preferences^48^, cognitive or demographic characteristics^49^, and community or cultural factors^50^. While studies have examined priming health goals^51^, personalization based on people’s health priorities represents a gap in the literature. Personalization incorporating personal priorities also presents an opportunity for interventions to be responsive to members of under-represented groups. For example, previous studies show that tailored healthy food labels and fruit and vegetable promotion posters that incorporated input from the communities in which they were to be implemented outperform non-tailored materials in a rural, minority population^50^, and in a culturally diverse school lunchroom setting^52^.

We estimate the impact of personalization based on individuals’ health priorities on the likelihood that participants chose a healthy food. We also performed an exploratory analysis of how each condition affected people’s satisfaction with the items they chose. We hypothesize that interventions designed to prime people to consider their health priority or that presented individuals with a subset of healthy foods matching their health priority would significantly increase the likelihood of people choosing a healthy food, but conditions that combined these two approaches would have an even greater impact on the choice of healthy foods.

## Methods

The research and analysis plans were pre-registered using the Open Science Framework registry (https://osf.io/86haq/?view_only=eb381abbf6214ef59e0a497c682c87cb). The University of Nebraska-Lincoln Institutional Review Board approved the research (IRB protocol #20221122409EX). All research was performed in accordance with relevant guidelines/regulations, including the Declaration of Helsinki. All participants provided written informed consent before beginning the research. Participants were adult (which is defined as ≥ 19 years of age by the US state in which the researchers work) residents of the US recruited from the Dynata consumer panel, with US Census-based demographic quotas employed to recruit a sample representative of the distribution of demographic characteristics in the US. The quotas set requirements for sex, age, income, and education characteristics.

In this research, we test the impact of personalizing dietary recommendations based on participants’ self-identified health priorities. We implement the personalized dietary recommendation by customizing the sets of foods that an individual faces during a food choice task based on their response to a question asking them to identify their top health priority from a list of four possibilities. This intervention combines two novel interventions: a health priority priming intervention, in which individuals identified their top health priority, and a choice environment intervention that simplifies identification of the healthy options in a realistically complex food retail environment. There is some evidence suggesting that goal priming—a concept related to priority priming—can positively impact food choices^53^, although effectiveness has been found to depend on factors such as salience of the prime^54^, and strength of the goal^51^. Priming consideration of a health priority is likely to be relevant to a greater percentage of the population than goal priming, as goal priming may target a goal—such as weight loss—that is not held by everyone. Customizing the choice environment to individuals’ priorities reduces costs related to searching through all available options to find options that are beneficial given the individuals’ priority. If an individual is uncertain about what foods they should look for given their prioritized health outcome, choice environment customization may address that barrier as well.

Because we examine an intervention comprising two elements: (1) having participants identify their top health priority and (2) using that priority to customize a set of products that are responsive to that priority, we include six conditions that allow us to separate the impacts of the multiple intervention components. We developed the experiment on food choices to study the impact of the interventions following previous research^55–58^. A survey on elements of the food choice process—including satisfaction with the foods that participants selected and demographic questions—followed the experiment on food choices. The question about choice satisfaction featured a five-point scale ranging from “Not at all satisfied” to “Completely satisfied.” Demographic questions included the respondent’s age (in categories ranging from “19–24 years of age” to “75 years of age or older”); gender (female, male, other); household income in 2023 (ranging from “Less than $25,000” to “$150,000 or more”); highest level of education the respondent had completed (from “Less than high school” to a “Graduate/Professional degree”); and race/ethnicity categories that are applicable in the US. All questions featured a “prefer not to answer” option. The experiment and survey were programmed in Qualtrics, a survey development software program^59^. All survey questions were included in the pre-registered materials (https://osf.io/86haq/?view_only=eb381abbf6214ef59e0a497c682c87cb).

### Intervention conditions

Participants were randomized to one of the six conditions: (1) a control condition (CTRL); (2) a health priority prime condition (HP); (3) a healthy product optional subset condition (HS); (4) a health priority prime question with optional healthy product subset condition (HP + HS); (5) a health priority prime question with optional subset described as being good for the health priority selected by the participant (HP + PSO); or (6) a personalized product set based on the participant’s selected health priority, with the option to view all products (PPS). Table 1 provides a description of each condition.

**Table 1:** Description of intervention conditions employed in the survey experiment.

Condition (Abbreviation)	Answered health priority question?	Received healthy product subset intervention?
Control (CTRL)	No	No
Health Prime (HP)	Yes	No
Healthy Subset (HS)	No	Yes—participants chose between viewing full set of products or a subset described as generally healthy, e.g., “Healthy snacks.”
Health Prime + Healthy Subset (HP + HS)	Yes	Yes—participants chose between viewing full set of products or a subset described as generally healthy, e.g., “Healthy snacks.”
Health Prime + Personalized Subset Option (HP + PSO)	Yes	Yes—participants chose between viewing full set of products or a subset described as meeting the individual’s health priority, e.g., “Snacks that are good for gut health.”
Personalized Product Set (PPS)	Yes	Yes—immediately viewed the subset of products described as meeting the individual’s health priority, e.g., “Snacks that are good for gut health,” with an option to view the full set of products

All conditions featuring a health priority prime (HP, HP + HS, HP + PSO, PPS) asked participants to indicate their top health priority from a list of four broad health priorities: cognitive health, gut health, metabolic health, or physical health. These priorities were selected because they constitute important elements of health that are linked to diet^5,60–72^. While the categories cover health issues like heart health, cancer, diabetes, and obesity that comprise many of the most deadly non-communicable diseases^73^, we also included health aims that surveys have shown to be motivating to the general public, such as attempts to improve one’s “shape”^66^. Each health priority category provided examples of specific outcomes that fall under the broader health priority. Table 2 shows the categories and examples presented in the research. While many of these conditions are interrelated—for instance, poor metabolic health outcomes are related to poor cognitive health outcomes—the purpose of the health priority identification task was to prime individuals to think about reasons why they cared about their health. In fact, all food options in the personalized product sets were identical for all health priorities to cleanly test the impact of the intervention.

**Table 2:** List of health priority options presented to individuals in the health priority prime conditions and examples of impacts.

Health priority	Examples
Cognitive Health	Improving concentration, memory, and function; reducing risk of dementia and Alzheimer’s disease
Gut Health	Improving regularity; prevention of bloating and gas
Metabolic Health	Improving heart health; reducing risk of cancer, diabetes, and obesity
Physical Health	Improving energy, physical performance; being in good shape

Next, all participants made a hypothetical food choice from three categories: snacks, soups, and frozen patties and burgers (FPB). To reduce hypothetical biases, participants read a cheap-talk script, which asked participants to approach the choices as though they were making an actual purchase, including considering other things they would spend money on. Cheap-talk scripts are a widely used method that have been found to significantly reduce hypothetical bias^75,76^.

In each category, participants selected one item that they would purchase but could also indicate that they would not purchase any of the available products. There were 30 products per category, with 10 of those products qualifying as healthy. All products listed information about calories, saturated fat, sodium, dietary fiber, added sugar, potassium, iron, and calcium, as well as price below the product image and name. This does not include all the nutrients that are mandatorily listed on nutrition facts panels (NFPs), which are printed on the back or side of food packages, in the US. Due to limitations with the software, we chose to include a subset of information, which included calories—the piece of information provided at the top of NFPs—and the relevant nutrients identified as nutrients of public health concern by US government agencies^75,76^. Since vitamin D is not naturally present in meaningful amounts in the foods in the study’s product categories, we instead included information about iron. Product prices were based on US retail prices in early 2024 at national retailers.

In the CTRL and HP conditions, participants viewed all products in a food category at once and then moved on to the next category. In the conditions containing a subset choice (HS, HP + HS, HP + PSO), participants had the option to view all 30 products in a category, or they could select to view a subset containing all the healthy items. If participants initially chose to view the healthy subset, they could return to the full set if they did not find anything of interest in the subset.

In the PPS condition, participants immediately viewed the healthy set of products, which was described as a set of products that were beneficial for their health priority. We used piped text to name the specific condition they had selected rather than simply saying “your health priority.” Participants in PPS could exit the personalized subset to view all available products (including unhealthy products) if they did not find anything of interest in the personalized subset. Participants made product set decisions in each food category—that is, they could choose to view the healthy subset in the snack category, but the full set of soups. After participants completed the food choices, they answered a series of survey questions. We collected data about participants’ satisfaction with the products they selected during the experiment and demographic characteristics, including sex, age, education, household income, and race/ethnicity. The choice satisfaction data were collected immediately after participants completed the food choice process. The choice satisfaction variable was collected for all products as a whole, rather than for each food category. Thus, we used a dataset featuring one observation per participant.

### Product set construction

We selected products in soup, snack, and FPB categories to have a range of nutritional profiles. We chose these three product categories because they include food products that feature a distribution of nutritional profiles rather than predominantly healthy (e.g., fresh produce) or unhealthy (e.g., desserts), so that there could be variation in the nutritional quality of products selected. Further, we chose product categories that have been linked with unhealthy dietary patterns. Burgers are one of the top sources of red meat consumption in the US^77^. High red meat consumption is linked with negative health outcomes^77^, and numerous health organizations recommend limiting its consumption^78–80^. Snacking, which in the literature is defined as food consumption outside of normal meal times^81^, has increased in the US in recent decades^81,82^, Most snacking consumption constitutes foods such as chips, crackers, candy, and other highly processed sweet or savory items^83^, and has been linked with higher consumption of calories and nutrients to avoid^81–83^. Soups are a major source of sodium, identified as a nutrient of public health concern^75^, in Americans’ diets^84^.

To construct the healthy subset, we used nutrient levels of four nutrients that have been identified as nutrients of public health concern in the Dietary Guidelines for Americans 2020–2025^75^; saturated fat, sodium, dietary fiber, and added sugar. Each of these nutrients has been associated positively (dietary fiber) or negatively (saturated fat, sodium, added sugar) with the four health priority options used in this study^5,65,68,69,71,85–88^. We established inclusion criteria based on recommended daily values for each of these nutrients to be considered a healthy food (Table 3). Due to a desire to maintain the same number of products in the healthy and full product sets for all product categories, we varied criteria for two nutrients across categories. Products that met the criteria for all nutrients were included in the healthy food subset, while products that did not meet at least one of the criteria levels were excluded from the healthy subset.

**Table 3:** Nutrient criteria levels per serving for a food to qualify as a healthy product.

Product category	Serving size	Dietary fiber	Saturated fat	Sodium	Added sugar
Healthy soups	240 g (as prepared)	≥ 10% DV: 2.8 g	≤ 20% DV: 4 g	≤ 30% DV: 690 mg	≤ 10% DV: 5 g
Healthy snacks	28 g	≥ 10% DV: 2.8 g	≤ 10% DV: 2 g	≤ 10% DV: 230 mg	≤ 10% DV: 5 g
Healthy FPB	120 g	≥ 10% DV: 2.8 g	≤ 20% DV: 4 g	≤ 30% DV: 690 mg	≤ 10% DV: 5 g

*DV* daily value, *FPB* frozen patties and burgers.

### Data

Data were collected in June 2024. Once data collection was complete, we downloaded the data from Qualtrics. Conditions were coded numerically in a non-sequential manner to blind the researcher conducting the data analysis and then decoded for reporting after we completed the analyses included in this paper. Food items were categorized as healthy or not, based on the criteria described above, resulting in a binary variable indicating the choice of a healthy food. To examine differences in nutrient levels across conditions, we used the numeric value listed on the products’ nutrition facts panel, with one exception: nutrients that were reported as < 1 unit of the nutrient (e.g., < 1 g dietary fiber) were coded as 0.5 units to enable numeric analysis. We analyzed additional elements beyond those used as inclusion criteria: Calories, saturated fat, added sugar, sodium, dietary fiber, iron, calcium, and potassium.

### Analysis

We used R Studio to analyze the data^89^. We tested for differences in demographic characteristics between the full sample and data from the US census^90^ using chi-squared tests, and among conditions using Fisher Exact tests. To analyze the impact of the intervention conditions on the likelihood that participants choose a healthy item in each category relative to the control condition, we conducted logistic regressions using the R package “MASS”^91^. We reported the analysis with two robustness checks. First, we reported the regression of the healthy food dependent variable on the five intervention conditions using the control condition as the reference condition. In the robustness checks, we included (1st) food category variables and (2nd) food category and demographic variables to test the robustness of the first analysis. We tested for differences in the estimated coefficients among intervention conditions with linear hypothesis tests using the R package “car”^92^. We reported adjusted odds ratios (aOR) and 95% confidence intervals (95% CI) for intervention variables full results are reported in online supplementary materials. To determine significant differences in nutrient contents of foods chosen among conditions, we used ANOVA followed by Tukey’s Honestly Significant Difference test.

Finally, we conducted an exploratory analysis of the impact of the intervention conditions on participants’ reported satisfaction with the choices they made, which was collected as an ordinal variable with five categories ranging from “not at all satisfied” to “completely satisfied.” In addition to the independent variables included in the analysis of food choice healthiness, we also included a variable to capture the number of products (excluding “I would not choose any”) an individual selected in the choice process, which could range from 0 to 3. We reported three analyses: the simple model featuring only the condition variables, a second version that added the variable indicating the number of items the individual selected from the three categories, and a third model that additionally included demographic variables using ordinal logistic regression in the R package “MASS”^91^. We reported aORs and 95% CI for independent variables.

## Results

**Fig. 1:**
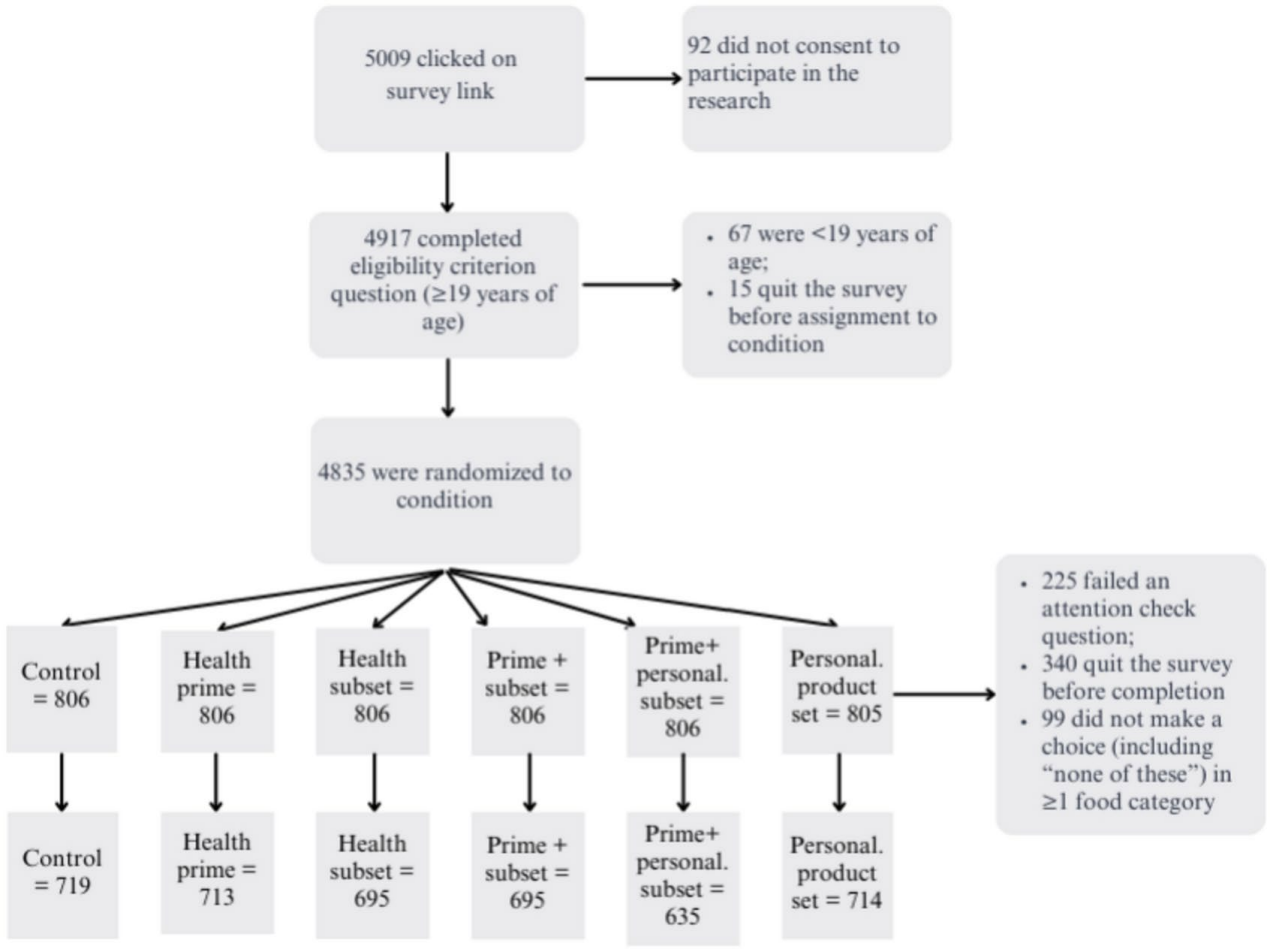
Flow chart of online research participants.

Figure 1 depicts information about participants in the survey. Just over 5000 individuals clicked on the survey link distributed by Dynata. After accounting for individuals who did not consent to the research, did not meet our eligibility criterion, quit the survey, failed an attention check, and/or did not respond to required questions, we ultimately received 4171 completed surveys (83.3% of those who initially clicked on the survey link).

**Fig. 2:**
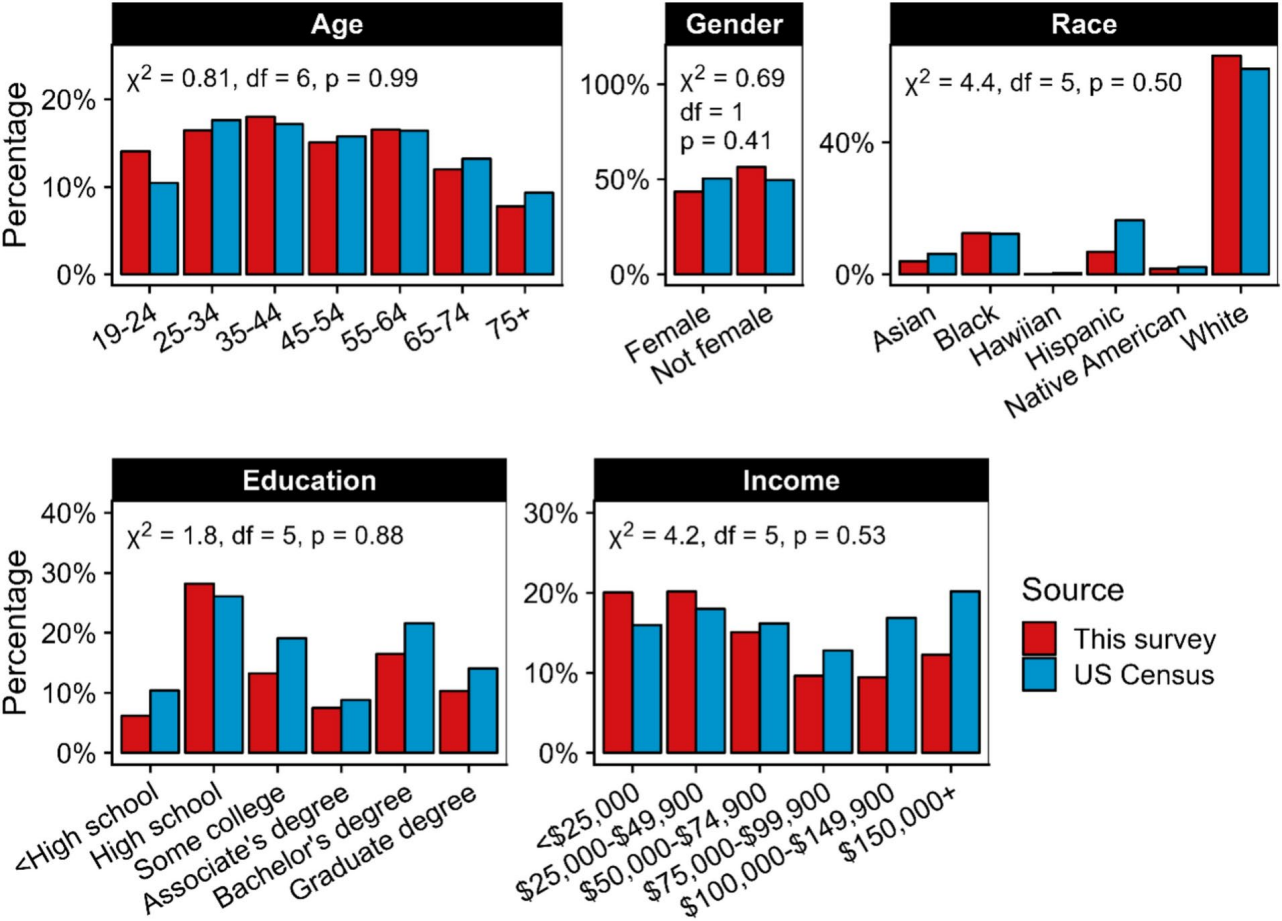
Demographics of the full sample of participants from this survey compared with US Census data.

Our final dataset comprised 12,513 observations of food choices, as each participant made three product choices. Figure 2 compares data on participant demographics for the full sample with the US population characteristics. The distribution of the full sample does not differ significantly from the US (adult) population. We also report the distribution of all demographic characteristics for the full sample and each experimental condition in online supplementary materials. None of the demographic characteristics differed significantly among conditions, suggesting that randomization was successful.

### Impact of interventions on choice of healthy foods

**Fig. 3:**
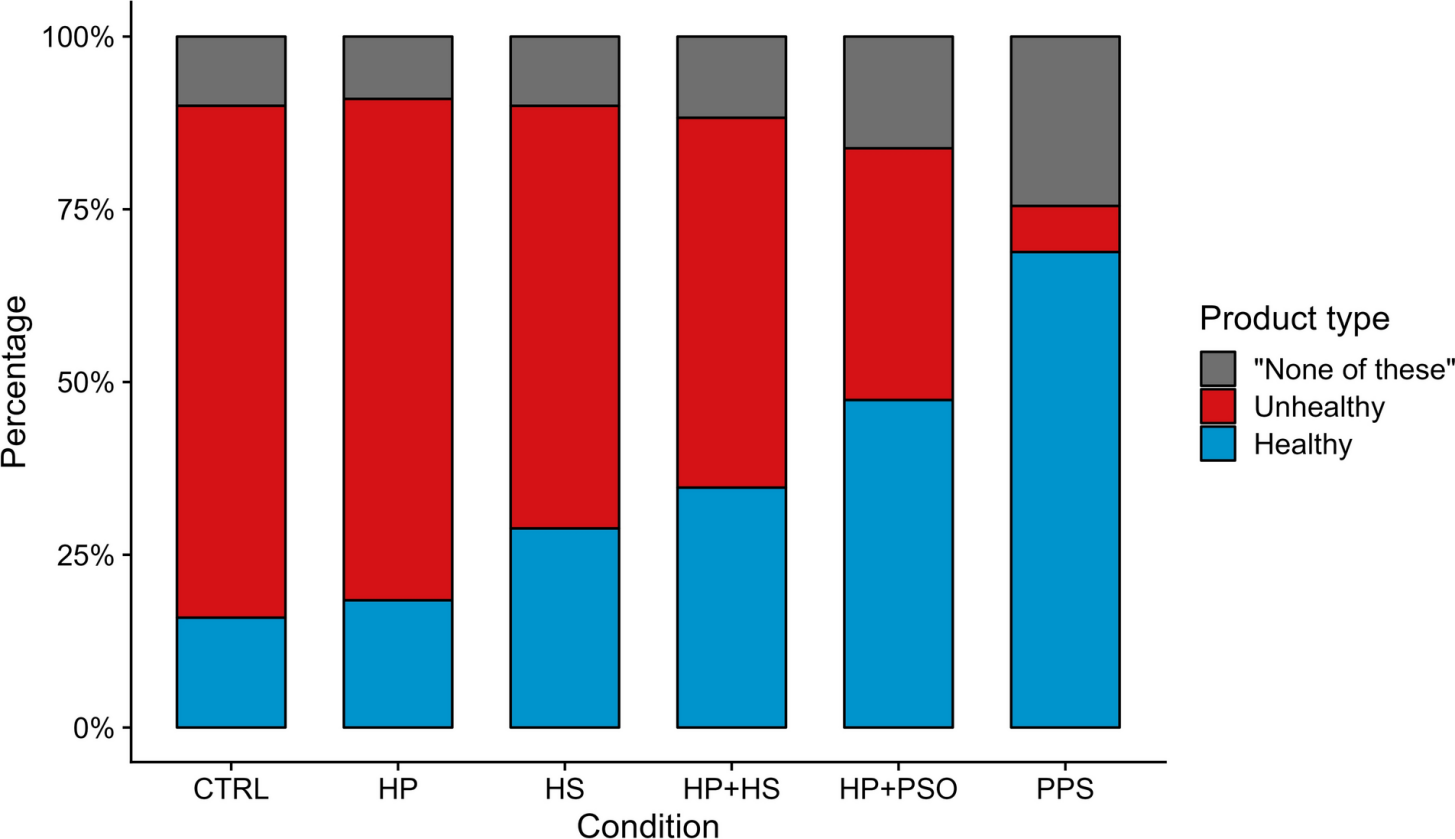
Percentage of healthy products, unhealthy products, and “none of these” selected by participants by condition.

The percentage of healthy products chosen ranged from 15.9% of choices in CTRL condition to 68.8% in PPS (Fig. 3). Selection of “none of these” also increased, particularly in the two conditions featuring personalization. Unhealthy options, on the other hand, decreased from 74.1% in CTRL to 6.7% in PPS.

The effect of the intervention conditions on the likelihood that participants selected a healthy food product is presented in Table 4. Column 1 displays results for the basic model with no additional control variables, while columns 2 and 3 add food category and then demographic variables. The ordering, sign, and significance of estimates was consistent across models, demonstrating the robustness of the relationships between treatment conditions and healthfulness of the foods chosen. Overall, HP had a significant but relatively small impact on the likelihood that participants chose a healthier product, making participants approximately 1.2 times more likely to choose a healthy food than in CTRL (*p* < 0.05). However, providing an easy way for participants to identify healthy products—even without priming their health priorities—led them to be approximately 2.2 times more likely to select a healthy item in HS than in CTRL. This estimate was significantly greater than CTRL and HP (*p* < 0.001, both comparisons).

**Table 4:** Adjusted odds ratios (aOR) and 95% confidence intervals (CI) for the impact of intervention conditions on the choice of a healthy food.

	1. aOR (95%CI): conditions	2. aOR (95% CI): conditions + food categories	3. aOR (95%CI): conditions, food categories, and demographics
Intercept	0.19 (0.17, 0.21)***	0.12 (0.10, 0.14)***	0.08 (0.06, 0.11)***
Health prime	1.19 (1.02, 1.40)*	1.20 (1.02, 1.41)*	1.18 (1.00, 1.39)*
Healthy subset	2.14 (1.85, 2.49)***	2.17 (1.87, 2.53)***	2.20 (1.89, 2.56)***
Health prime + healthy subset	2.81 (2.43, 3.26)***	2.88 (2.48, 3.34)***	2.87 (2.48, 3.34)***
Health Prime + personalized subset	4.77 (4.12, 5.52)***	4.95 (4.27, 5.74)***	4.98 (4.29, 5.79)***
Personalized product set	11.67 (10.08, 13.54)***	12.42 (10.71, 14.44)***	12.81 (11.03, 14.92)***
Food category control variables	No	Yes	Yes
Demographic control variables	No	No	Yes
Akaike information criterion	14,437	14,157	14,017

Data from experiment and survey. N = 12,513. *** = p < 0.001; * = p < 0.05.

The remaining conditions constitute combined interventions. The HP + HS condition (combining the health prime with the healthy product subset) increased the likelihood that participants chose a healthy item by around 2.8 times relative to CTRL. This effect was significantly greater than CTRL, HP, and HS (*p* < 0.001, all comparisons). People in the HP + PSO condition, which combined the health priority prime with the subset of products identified as meeting the individual’s health priority, were 4.8 to 5 times more likely to select a healthy food than those in CTRL. Again, this estimate was significantly greater than all preceding conditions (*p* < 0.001, all comparisons).

In the PPS condition, personalization of the food choice environment based on an individual’s self-identified health priorities led to a likelihood of selecting a healthy food that was 11.7 to 12.8 times greater than in CTRL. This estimated effect is significantly greater than all other conditions (*p* < 0.001, all comparisons).

### Impact of interventions on nutrient content of foods chosen

**Fig. 4:**
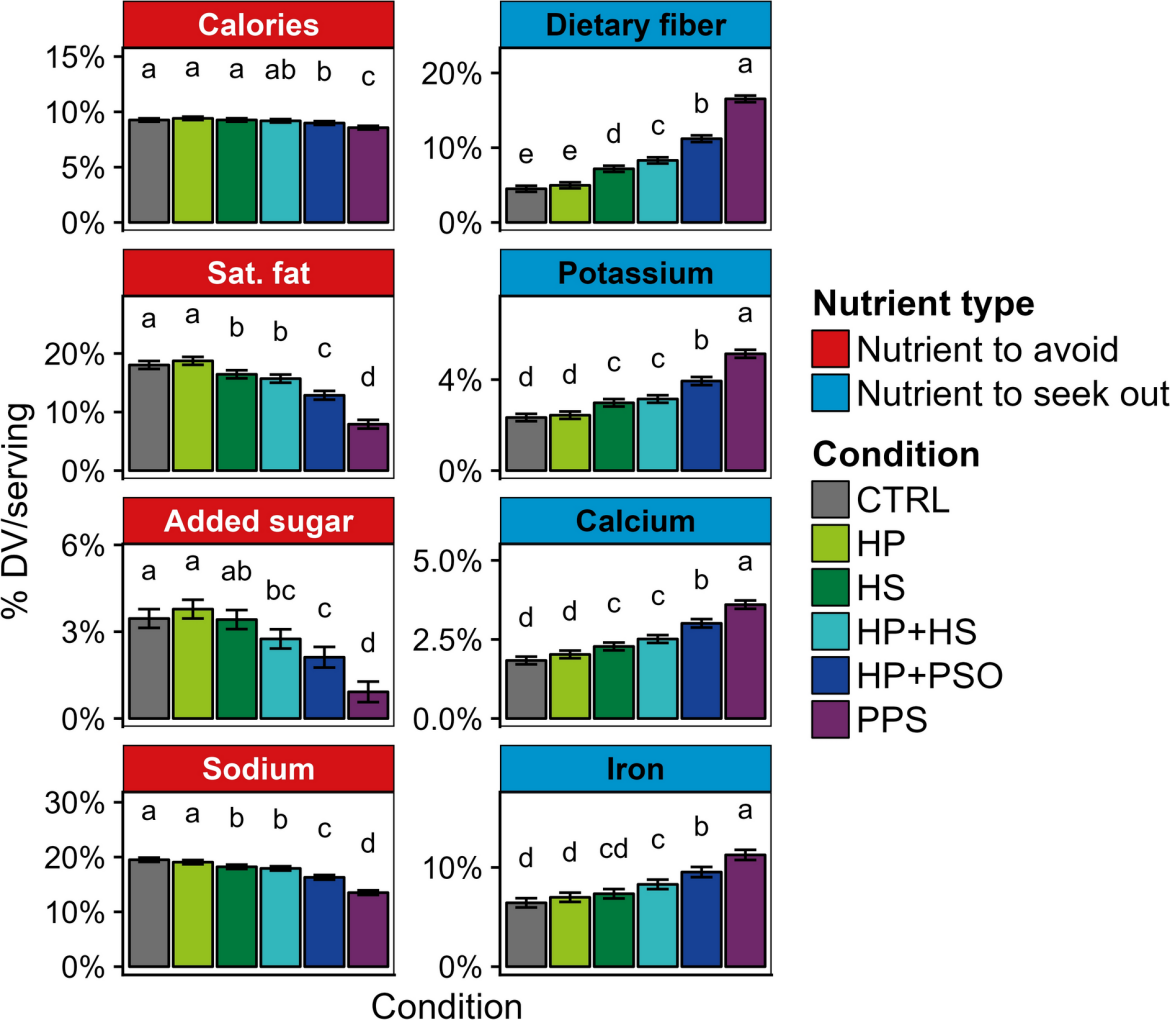
Nutrient content of foods selected by participants by condition. Bars represent the means; error bars show the 95% confidence intervals; Sat. fat = saturated fat; DV = Daily Value; bars marked with different lower-case letters are significantly different within nutrient (Tukey’s Honestly Significant Difference test, *p* < 0.05).

To validate the findings in Table 4, we examined the nutrient contents of foods chosen by condition. The HP condition did not significantly improve nutrient content of the foods selected (Fig. 4). The HS condition decreased sodium and increased dietary fiber, potassium, and calcium relative to the control, while the HP + HS condition additionally decreased saturated fat and added sugar while increasing iron. Figure 4 shows the remarkable effect of the conditions that included a personalized subset of products on the nutrient content of foods selected. When participants were given the option to view a subset of products based on their health priority (HP + PSO), the contents of all nutrients that are generally recommended to limit in healthy diets (i.e., saturated fat, added sugar, and sodium) were significantly lower than all conditions not containing a personalized subset, while nutrients that are recommended to seek out were significantly increased (i.e., dietary fiber, potassium, calcium, and iron). When participants immediately viewed the personalized subset (PPS), all nutrients to avoid were significantly lower than in all other conditions, while nutrients that are recommended to seek out were significantly increased.

### Exploratory analysis of the effect of interventions on satisfaction with food choices

As was shown in Fig. 3, an increasing proportion of participants selected “None of these” in the combined conditions. This prompted us to conduct an exploratory analysis of subjects’ satisfaction with their product choices, assuming that an increasing proportion of panelists not making any choice may indicate less satisfaction with products chosen in that condition. Table 5 shows that participants in conditions featuring a personalized subset were significantly more likely to report a lower level of satisfaction than participants in the control condition. However, when we accounted for the number of products each participant chose, only participants in the PPS condition were significantly more likely to report lower levels of satisfaction. Finding a product that they wanted to purchase significantly impacted participants’ reported satisfaction with the choice made—for each additional item selected, participants were 2.7 times more likely (95% CI = [2.48, 2.96]) to report higher satisfaction with the choices they made. The addition of demographic variables did not change the significance of the conditions.

**Table 5:** Adjusted odds ratios (aOR) and 95% confidence intervals (CI) for the impact of intervention conditions on satisfaction with food choices.

	1. aOR (95% CI): conditions + food categories	2. aOR (95% CI): conditions, food categories, products chosen	3. aOR (95%CI): conditions, food categories, products chosen, and demographics
Health prime	0.99 (0.82, 1.21)	0.96 (0.79, 1.16)	0.94 (0.78, 1.15)
Healthy subset	1.08 (0.89, 1.32)	1.08 (0.89, 1.32)	1.07 (0.88, 1.31)
Health Prime + healthy subset	0.87 (0.72, 1.06)	0.93 (0.76, 1.13)	0.93 (0.76, 1.13)
Health Prime + personalized subset	0.82 (0.67, 1.00)	0.94 (0.77, 1.15)	0.92 (0.75, 1.12)
Personalized product set	0.51 (0.42, 0.63)***	0.71 (0.58, 0.87)***	0.70 (0.57, 0.86)***
Number of products chosen	–	2.71 (2.48, 2.96)***	2.67 (2.45, 2.92)***
Product category Variables	Yes	Yes	Yes
Demographic Control variables	No	No	Yes

Data from experiment and survey. N = 4,171. ***p < 0.001.

## Discussion

We report the results of an experiment on a novel form of dietary personalization: providing personalized product sets during food choice based on individuals’ self-reported health priorities. We incorporated additional conditions to disentangle the distinct contributions of the personalized product set. Although all intervention conditions increased healthy food choices, the PPS substantially increased the likelihood that individuals selected a healthy item, relative both to the control condition and all other intervention conditions. An interesting finding that illustrates the potential impact of personalization is that the only difference between the HP + HS and the HP + PSO conditions was the naming of the subset. In the first case, it was described as a healthy subset, whereas the second condition described it as responsive to the individual’s health priority. This small change yielded a significant increase in the likelihood that a healthy item was chosen (2.8 versus 4.8, respectively, *p* < 0.001). The changes in food choices correspond to significant improvements in the nutrient profile of foods selected. The analysis focused on nutrients identified as nutrients of public health concern in the Dietary Guidelines for Americans, 2020–2025, including nutrients that are over-consumed, such as saturated fats, sodium, and added sugars, as well as nutrients that are under-consumed, including calcium, dietary fiber, and potassium. However, an exploratory analysis of participants’ satisfaction with product choices suggests that providing a personalized product set may reduce satisfaction, which will be discussed more below.

The PPS condition addresses three important potential barriers to healthy choices: lower relative attention to future versus current outcomes^93^, search costs associated with complex, multi-item choice environments^13^, and knowledge or belief-related barriers to identification of healthy food options^93^. Reducing these barriers to search/identification of healthy food further aligns individuals’ health priorities with actions tied to those priorities.

Research has documented impacts of each of these factors on health behaviors. There is growing evidence that people more naturally consider current than future outcomes during decision-making. Many people appear to overlook future impacts of current decisions^93^, which can prevent their preferences for the distribution of benefits through time from being expressed in their immediate actions^94^. In the food context, people who habitually consider future consequences make healthier choices^95^. Actively considering health—a delayed outcome of food choice—during a specific instance of choice also leads to healthier food choices^96^, and there is evidence that active consideration of health—and healthier choices—can be prompted through simple health reminder messages^56,97–99^. However, taste attributes—an immediate outcome of food choice—appear to be assimilated into decision processes more quickly than health attributes^100^.

Our findings corroborate findings from previous studies. For instance, the small impact of the simple health priority prime reflects mixed results from previous literature on goal-priming^13,14,101^. It may also echo the importance of addressing the complexity of the choice environment. Studies conducted in a supermarket in a rural, low-income minority community, online among a general adult US population, and in a virtual reality environment all provide evidence that the ease of finding healthy foods affects the likelihood that healthy foods are chosen^13,14,101^. Research varying the convenience of lower/higher calorie foods found that making higher calorie options less convenient decreased calorie content of foods selected by fast food customers^102^.

There is substantial evidence that knowledge and/or beliefs about foods and nutrition play an important role in shaping food choices. People with higher levels of nutritional knowledge make healthier choices^11,103^. Beliefs, on the other hand, can promote or inhibit healthier choices. Beliefs that healthy behaviors are less likely to yield positive outcomes inhibit those behaviors^104^, while beliefs about the health of certain diets—such as the gluten-free diet—affect the likelihood that people decide to follow that diet^104^. Further, there is evidence that many people hold inaccurate beliefs about the food attribute, such as calories^105^, sodium^106^, and fat^107^, relationships between food price and health^108^, or do not understand links between nutrients, like dietary fiber, and health outcomes^10^, which affects their likelihood of seeking out fiber information during food choice^109–111^. However, there is evidence that objective information^109–111^, especially when made salient via reminder messages^27,57^, can correct misperceptions.

While the PPS condition significantly—and markedly—increased the choice of healthy foods, participants in this condition were also less likely to be satisfied with their product choices than participants in other conditions. This was potentially driven by the fact that participants in the PPS condition chose fewer products than in most other conditions. As depicted in Fig. 3, the percentage of participants selecting an unhealthy food in the PPS condition decreased markedly relative to other conditions, which mirrors results from studies examining food choice and consumption behavior when healthy food materials are customized to the community or individual^112^. Research on choice satisfaction indicates that when measured before food consumption occurs, satisfaction relates to the anticipated experience of consumption as well as desires^112^. This has led some researchers to propose using a promotional strategy for healthy foods that highlights the pleasure of consuming those foods^113^. Indeed, a study of the fruit and vegetable consumption of older Italian adults focused on health involvement and satisfaction with fruit and vegetable consumption found that higher satisfaction was the most significant predictor of consumption^114^. In the current study, only ten healthy items were available per product category, which may have limited participants’ ability to find preferred foods.

### Limitations

There are limitations that need to be acknowledged. First, we offered a pre-set selection of four over-arching health priorities—with sub-examples for each priority—for participants to consider. Not being able to fully specify one’s health priority may have diminished participants’ engagement with the intervention conditions’ attempts to personalize the choice environment, suggesting that the estimated impacts of the personalization conditions may be conservative. In future work, we hope to be able to increase the sophistication of the programming that would allow linking unconstrained expression of individuals’ health priorities with responsive food suggestions.

Second, the decisions made in the experiment are hypothetical, which was necessitated by a desire to recruit a large sample of respondents with diverse demographic characteristics from throughout the US. While we employed techniques meant to mitigate hypothetical bias, we cannot rule out the possibility that choices would differ if participants were exchanging real money for real products.

Third, we examined choices in a limited number of food product categories. The impact of the intervention on the healthfulness of choices that participants made in the frozen patties and burgers, snacks, and soups categories may not reflect the impact that the intervention would have in other categories. As noted above, we chose product categories that included food products featuring a distribution of food product healthiness (rather than uniformly healthy or unhealthy, which would by default limit behavioral variation in healthy food choice)^77^. Further, we chose product categories that have been linked with unhealthy dietary patterns. Burgers are one of the top sources of red meat consumption in the US^77^, and previous research on choice of foods with pulse-based plant proteins versus foods with animal-source proteins showed that people were much more likely to select an animal product in the frozen patties and burgers category than in frozen dinners and entrees^81–83^, making the inclusion of this category a more conservative test of the intervention conditions. Snacks, which feature many highly processed products, have been linked with higher consumption of added sugars and are a significant source of calories^81–83^. Soups are a major source of sodium, which is a nutrient of public health concern due to overconsumption^75^, in Americans’ diets^84^.

Fourth, the presentation of nutrition information does not precisely reflect real-world conditions. Due to programming limitations, we included nutrition information directly under the product name and image rather than having participants access it through a link. In real-world retail environments, consumers must seek out that information (by examining the side or back of the product in physical stores or clicking a link to the information or scrolling to the information in online retail sites). This may have increased the likelihood that people used nutrition information^115^. Second, because we were displaying nutrient information immediately below the product, we only included partial nutrient information. We prioritized nutrients of public health concern (sodium, saturated fat, added sugar, dietary fiber, calcium, and potassium)^75^, though we also included calories and iron. These include the top three pieces of nutrition information that a survey identified as influencing consumers’ beliefs about what differentiates healthy from unhealthy foods^116^. However, some individuals may have liked to have used other pieces of nutrition information that were not included in the study. Both the presentation and composition of nutrition information may have influenced decisions, potentially altering participants’ choices relative to a real-world choice environment. We will address this limitation in future research.

An additional limitation is that this study examined one choice event, whereas the health impacts of food choices accrue from numerous, repeated consumption decisions. It is not clear how people would respond to the studied intervention throughout time. We are unaware of long-term research in the food domain on interventions that facilitate consideration and identification of health-promoting foods. However, in a different health domain, interventions reminding people with gym memberships to go to the gym have demonstrated efficacy over a months-long intervention, with evidence of continued impact even after the intervention ended^117,118^. Critically, more research is needed to understand the potential of the intervention studied in this paper to effectively promote healthier choices in real-world settings over long periods of time.

## Conclusions

An intervention that connects people’s health priorities to a responsive action—foods that would help achieve those priorities—may promote healthy choices. However, strategies to support people’s satisfaction with their healthier choices need further examination. The rise in online shopping and powerful smartphones could create opportunities to personalize information or food options to individuals’ unique priorities or goals.

## Data Availability

Data and materials are available at osf.io/86haq.
